# Ovarian cancer risk reduction by salpingectomy during non-gynaecological surgery: scoping review

**DOI:** 10.1093/bjsopen/zrae161

**Published:** 2025-01-28

**Authors:** Charlotte Fisch, Malou E Gelderblom, Rosella P M G Hermens, Philip R de Reuver, Simon W Nienhuijs, Diederik M Somford, Joanne A de Hullu, Jurgen M J Piek

**Affiliations:** Department of Obstetrics and Gynecology, Radboud University Medical Centre, Nijmegen, The Netherlands; Department of Obstetrics and Gynecology, Radboud University Medical Centre, Nijmegen, The Netherlands; Department of IQ Health, Radboud University Medical Centre, Nijmegen, The Netherlands; Department of Surgery, Radboud University Medical Centre, Nijmegen, The Netherlands; Department of Surgery, Catharina Hospital, Eindhoven, The Netherlands; Department of Urology, Canisius-Wilhelmina Hospital, Nijmegen, The Netherlands; Department of Obstetrics and Gynecology, Radboud University Medical Centre, Nijmegen, The Netherlands; Department of Obstetrics and Gynecology, and Catharina Cancer Institute, Catharina Hospital, Eindhoven, The Netherlands

## Abstract

**Background:**

Ovarian cancer is the leading cause of death among gynaecological cancers. The identification of the fallopian tube epithelium as the origin of most ovarian cancers introduces a novel prevention strategy by removing the fallopian tubes during an already indicated abdominal surgery for another reason, also known as an opportunistic salpingectomy. This preventive opportunity is evidence based, recommended and established at the time of gynaecologic surgery in many countries worldwide. To expand interest among surgeons in performing a salpingectomy during non-gynaecological surgery, the aim of this review is to identify knowledge gaps during those surgeries.

**Methods:**

A scoping review was performed following the PRISMA-Scoping Review (ScR) checklist. PubMed, Embase, Web of Science, Cumulative Index to Nursing and Allied Health Literature (CINAHL) database and Cochrane Library were systematically searched from inception to November 2024. Trial registers were searched for ongoing trials. All studies reporting original data on salpingectomy during non-gynaecological surgery were included. Outcomes were provided narratively.

**Results:**

Eighteen studies were identified reporting on the implementation, surgical feasibility, patients’ perspectives, physicians’ knowledge and cost-effectiveness of an opportunistic salpingectomy during non-gynaecological surgery. Population-level data indicate that an opportunistic salpingectomy is rarely performed in non-gynaecological surgeries. High success rates and no complications of an opportunistic salpingectomy were observed during bariatric surgery and cholecystectomies. However, performing an additional salpingectomy appeared more time-consuming. Patients had strong interest in information on and willingness to undergo opportunistic salpingectomy. Cost-effectiveness analysis encourages opportunistic salpingectomy use, as models show reduced ovarian cancer incidence and mortality rate while being cost-effective.

**Conclusions:**

Opportunistic salpingectomy during non-gynaecologic surgery appears to be a promising method to prevent ovarian cancer. Implementing such a strategy will require education of multiple surgical disciplines, training and resolution of organizational issues.

## Teaser

This study explores the feasibility, patient interest and cost-effectiveness of opportunistic salpingectomy during non-gynaecological surgeries as a preventive measure for ovarian cancer. Thirteen included studies highlight its low implementation rates but demonstrate high success rates and patient willingness, particularly in procedures like bariatric surgery and cholecystectomies. Despite time constraints, the procedure shows promise in reducing ovarian cancer incidence and mortality rate, advocating for its consideration in non-gynaecological surgical settings.

## Introduction

Ovarian cancer (OC) is the most lethal gynaecological cancer and is the fifth most common cause of cancer-related deaths among women^1^. Additionally, approximately 80% of OCs are detected at an advanced stage due to late onset of non-specific symptoms^1,2^. Efforts to detect OC at an earlier stage in the asymptomatic population (with ultrasound and/or Ca125) have proven to be ineffective^3^. Generally, OC is treated aggressively with a combination of debulking surgery and chemotherapy. However, the majority of patients will develop recurrent disease and chemotherapy resistance, resulting in a poor 5-year survival rate of roughly 29%^1,2^. Despite evolution in the field of cancer therapy in general, the survival of OC has improved only very modestly over the past decades. This is partly attributed to tumour heterogeneity among OCs, which complicates the development of targeted therapies^4,5^. Due to the absence of effective screening programmes combined with limited treatment options, attention should be directed towards primary prevention.

Twenty years ago, a fundamental insight regarding the origin of most OCs was discovered. Preneoplastic lesions in the fallopian tube, known as serous tubal intraepithelial carcinomas (STICs), were identified as precursor lesions of high-grade serous OC^6–8^. As a consequence, the fallopian tube was indicated as the tissue of origin of the majority of OCs instead of the ovary^9^. This hypothesis brought forward the concept of surgical prevention through removal of the fallopian tubes as a concomitant procedure, a so-called opportunistic salpingectomy (OS), leaving the ovaries *in situ* to preserve female hormone production^10^.

Several retrospective cohort studies showed a risk reduction of 42–65% of OC after fallopian tube removal^11–14^. Furthermore, OS appears to be a safe procedure during gynaecological surgery without increasing the number of surgical complications and only increasing surgical time by minutes^15–19^. Consequently, OS is recommended in evidence-based guidelines by an expanding number of gynaecological societies^20–22^. The uptake at the time of gynaecologic surgery is established in a significant number of countries, although it varies significantly across racial and ethnic groups, as well as between and within countries^20,23–25^. However, the majority of surgeries performed in potentially eligible women are non-gynaecological abdominal surgeries. These non-gynaecological abdominal procedures present a new window of opportunity for the prevention of OC.

Therefore, this scoping review aims to identify knowledge gaps and to define research priorities needed for implementation of OS in non-gynaecological surgery.

## Methods

A scoping review was performed to investigate current available literature regarding OS during non-gynaecological surgery. The search was broad for literature that reported on implementation, surgical feasibility, eligible patient groups, patients’ perspectives, physicians’ perspectives, physicians’ knowledge and the cost-effectiveness of OS during non-gynaecological surgery.

This scoping review was reported according to the Joanna Briggs Institute (JBI) methodology and the PRISMA-Scoping Review (ScR) criteria for scoping reviews^26,27^ (*Supplementary material A*).

### Search strategy and selection criteria

#### Search strategy

The PubMed, Embase, Web of Science, Cumulative Index to Nursing and Allied Health Literature (CINAHL) database, the Cochrane Library and Trial registers (clinicaltrials.gov and International Clinical Trials Registry Platform [ICTRP]) were systematically searched in consultation with a medical librarian from inception to 7 November 2024 (see *Supplementary material B* for the full search strategy). The search string included medical subject headings, keywords and synonyms related to salpingectomy. Terms on tubal surgery and sterilization were added since this could include removal of the fallopian tubes. Moreover, reference lists and citations were manually reviewed to identify additional studies eligible for inclusion. In addition, the corresponding authors of ongoing clinical trials were contacted by e-mail for preprints.

#### Study selection

All conference abstracts and full publication studies describing original data about OS during non-gynaecological intra-abdominal surgery were considered for inclusion. Review studies, Letters to the Editor, correspondence and commentaries were excluded. Studies were further excluded if there was no full-text available, or if there was no availability in the English language. When a conference abstract was included, screening was done for a corresponding full-text publication. If a full-text article was available, the conference abstract was then excluded. In case of multiple publications describing the same data and methods, only the largest cohort was included.

#### Screening process

**Fig. 1:**
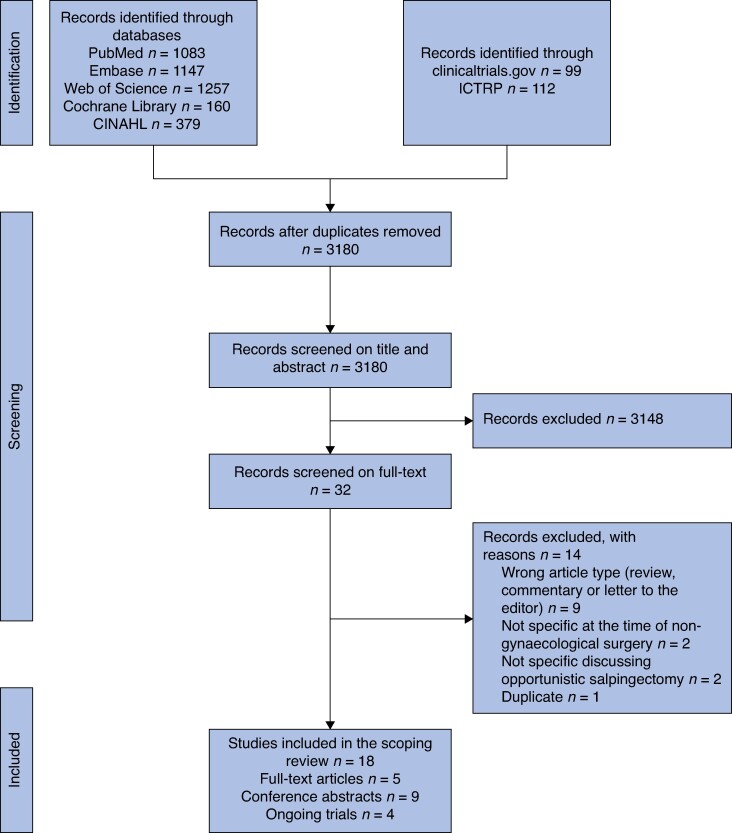
Study selection flow diagram CINAHL, Cumulative Index to Nursing and Allied Health Literature; ICTRP, International Clinical Trials Registry Platform.

Studies identified by the search were first imported into Endnote^TM^ 21 for deduplication. After deduplication, all remaining studies were imported to Covidence®, a web-based software platform (Covidence, Melbourne, Australia) for a second round of deduplication and subsequent screening. Two reviewers (C.F. and M.G.) independently assessed titles and abstracts of all identified studies according to the inclusion criteria. Potentially relevant studies were retrieved in full text and further assessed. Disparities regarding inclusion were resolved by consulting a third reviewer (J.P.). The flow diagram outlines an overview of the screening process for both published and registered clinical studies (*Fig. 1*).

### Data collection and analysis

Data were systematically extracted using an extraction table developed by the research team (*Supplementary material C*). Manuscript characteristics of interest were: first author, country of the study, publication year, study design, sample size, population characteristics and study outcomes (for example success rates of OS, duration of OS, patients’ and physicians’ perspectives and cost-effectiveness). Data were extracted by two independent researchers (C.F. and M.G.). Discrepancies were resolved through discussion with a third reviewer (J.P.). Outcomes were provided narratively and were grouped by outcome.

## Results

### Study selection

A total of 4237 studies were retrieved. After deduplication, 3180 studies were screened on title and abstract, of which 32 were retrieved in full text. Eighteen studies met the inclusion criteria (*Fig. 1*). No additional eligible studies were identified by manually examining the reference and citation lists of included studies, along with searching through conference abstracts for subsequently published articles.

### Included studies

Five studies were published in full text and nine as an abstract. Included studies reported on the implementation (*n* = 4)^28–31^, the surgical feasibility (*n* = 6)^32–37^, patients’ perspectives (*n* = 3)^34,38,39^, physicians’ knowledge (*n* = 2)^40,41^ and the cost-effectiveness of OS during non-gynaecological surgery (*n* = 4)^42–45^. No studies reporting on physicians’ perspectives were available, nor was there any study discussing the selection of eligible patients. *Table 1* shows a summary of all included studies.

**Table 1:** Summary of included studies arranged by core theme

	Authors and year	Title	Country of origin	Study design	Aim
Implementation	Cook and Landen^28^ 2020	Opportunistic salpingectomy in women undergoing non-gynaecological abdominal surgery	USA	Institutional review (conference abstract)	To estimate the impact that OS during non-gynaecological procedures would have on the incidence of epithelial ovarian cancer (EOC)
	Cathcart *et al*.^29^ 2023	Opportunistic salpingectomy during non-gynaecological surgery in the USA: a population-based retrospective study	USA	Population-based, retrospective, cross-sectional observational study	To estimate the incidence of OS during non-gynaecological surgery at a population level in the USA
	Cathcart and Harrison^31^ 2024	Opportunistic salpingectomy during non-gynaecologic ambulatory surgery in the USA, 2016–2020	USA	Population-based, retrospective, cross-sectional observational study	To evaluate the incidence of OS during non-gynaecologic ambulatory surgery in the USA
Surgical feasibility	Williams^32^ 2018	Concurrent bilateral salpingectomy for sterilization at the time of bariatric surgery	USA	Cohort study (conference abstract)	To demonstrate the safety and feasibility of performing bilateral salpingectomy for sterilization at the time of bariatric surgery
	Tomasch *et al*.^34^ 2020	Prophylactic salpingectomy for prevention of ovarian cancer at the time of elective laparoscopic cholecystectomy	Austria	Cohort study	To evaluate the feasibility and short-term complications of prophylactic salpingectomy in patients ≥ 45 years undergoing non-emergency laparoscopic cholecystectomy for benign indications
	Sagmeister *et al*.^33^ 2023	Might prophylactic salpingectomy be possible during bariatric surgery? (Can we see the tubes?)	Austria	Pilot study (conference abstract)	To evaluate whether the fallopian tubes could be visualized during bariatric surgery
Patients’ perspectives	Tomasch *et al*.^39^ 2018	Would women accept opportunistic (prophylactic) salpingectomy at the time of non-gynaecological surgery to prevent development of ovarian cancer?	Austria	Qualitative interview study	To assess whether it would be worthwhile to plan a trial offering salpingectomy at the time of laparoscopic cholecystectomy
	Tomasch *et al*.^34^ 2020	Prophylactic salpingectomy for prevention of ovarian cancer at the time of elective laparoscopic cholecystectomy	Austria	Cohort study	To evaluate the feasibility and short-term complications of prophylactic salpingectomy in patients ≥ 45 years undergoing non-emergency laparoscopic cholecystectomy for benign indications
	Cook *et al*.^38^ 2021	Patient attitudes toward opportunistic salpingectomy in non-gynaecological surgery	USA	Survey study (conference abstract)	To assess the interest of patients undergoing scheduled laparoscopic abdominal surgery for non-gynaecologic indications towards OS
Physicians’ knowledge	Bellamy *et al*.^40^ 2017	Opportunistic salpingectomy: a survey of knowledge and attitudes in healthcare professionals	UK	Survey study (conference abstract)	Evaluate knowledge and attitudes of healthcare professionals to OS
	Sussman *et al*.^41^ 2018	To oophorectomy or not to oophorectomy: practice patterns among urologists treating bladder cancer	USA	Survey study	To assess the knowledge base and current practice patterns of urologic oncologists regarding management of the gynaecological organs at the time of radical cystectomy
Cost-effectiveness	Hughes *et al*. ^42^ 2022	Opportunistic salpingectomy at the time of non-gynaecological elective procedures could reduce ovarian cancer-related costs and mortality rate	USA	Cost-effectiveness analyses	To determine the cost-effectiveness of OS and oophorectomy during non-gynaecological laparoscopic procedures and their effects on ovarian cancer mortality rate
	Matsuo *et al*.^43^ 2023	Opportunistic salpingectomy at the time of laparoscopic cholecystectomy for ovarian cancer prevention: a cost-effectiveness analysis	USA	Cost-effectiveness analyses	To perform a cost-effectiveness analysis to examine the utility and effectiveness of OS performed at the time of elective laparoscopic cholecystectomy
	Kather *et al*.^45^ 2024	Opportunistic salpingectomy for reducing the risk of ovarian cancer: a comprehensive decision analytic model for clinical and cost-effectiveness in gynecological and general abdominal surgery	Germany	Cost-effectiveness analyses	To evaluate the cost-effectiveness of OS in Germany
	Adjeia *et al*.^44^ 2024	Opportunistic salpingectomy during gynaecologic and non-gynaecologic abdominopelvic procedures for ovarian cancer primary prevention: a cost-effectiveness analysis	USA	Cost-effectiveness analyses	To evaluate the cost-effectiveness and impact of OS during six common abdominopelvic procedures on OC costs and overall survival

OS, opportunistic salpingectomy.

### Implementation

The implementation of OS during non-gynaecological surgeries was investigated in three studies^28,29,31^ and one clinical trial is registered^30^. In one study, the eligibility of patients aged between 35 and 75 years old for OS during three common abdominal surgeries (cholecystectomy, hernia repair surgery and bariatric surgery) was assessed^28^. Of 604 screened medical records, a total of 341 patients (56%) were found eligible for OS during surgery. The other 263 patients (44%) would potentially not be eligible for OS due to history of tubal sterilization or hysterectomy, although many of these may not have had a salpingectomy previously. This data was extrapolated to the number of annually performed cholecystectomies, hernia repair surgeries and bariatric surgeries in the USA. Consequently, approximately 400 000 patients would be eligible for OS per year. If OS was performed in these eligible patients, this could lead to an annual reduction of OC incidence between 28% and 38.5% (assuming the risk of OC is 1.7% and a risk reduction of 65% following OS). Therefore, OS would potentially prevent 3925 to 5394 OC cases annually in the USA. Cathcart *et al.*^29,31^ conducted a retrospective assessment from 2016 to 2020 investigating the incidence of OS during non-gynaecological surgery at a population level in the USA. The analysis identified 735 OS procedures in patients with a median age of 37 (interquartile range 34–43) years, hence OS was performed in less than <0.0001% of the non-gynaecological surgeries^29^. Surgeries comprised bowel resection or colostomy surgery (27.2%), breast surgery (8.2%), cholecystectomy (7.5%), bariatric surgery (29.2%) and hernia repair surgery (19.7%)^29^. The majority were elective procedures (85.7%). A laparoscopy was the most common surgical technique performed (54.4%), followed by a laparotomy (31.3%) and a robotic-assisted laparoscopy (14.3%). A subsequent study by the same authors^31^, which included the same retrospective data set, showed that the incidence of OS during non-gynaecologic procedures increased by 2.6% per quarter over the interval from 2016 to 2020. One registered trial (NCT04176484) is investigating the feasibility of OS during non-gynaecological surgery^30^. Part one of this clinical trial has already been completed and will be discussed later in the patients’ perspectives section^38^. The recruitment state of part two is, however, unknown as the clinical trial register has not been updated. *Table 2* shows an overview of the registered trials.

**Table 2:** Registered clinical trials

Title	Sponsor	Study type	Targeted no. of patients	Recruitment status	Primary outcome(s)	Clinical trials.gov identifier
Multi-disciplinary approach to the opportunistic salpingectomy for primary prevention of ovarian cancer^36^	Memorian Sloan Kettering Cancer Center	Interventional	400	Recruiting study (completion estimated in 2028)	Performance rate of OS among non-gynaecologic surgical patients (aged ≥45 years) Acceptance of OS among non-gynaecologic surgical patients (aged ≥45 years) after completion of an educational module	NCT06312124
Feasibility of opportunistic salpingectomy during non-gynecological surgery^30^	University of Virginia	Observational	150	Part 1: completed^38^ Part 2: unknown status (completion date unknown)	Part 1: feasibility and acceptability from patients (above 18 years) of OS based on survey response Part 2: the frequency and types of conditions that hinder the ability to perform an OS (patients above 25 years) to determine how frequently the eligible population could receive the suggested procedure	NCT04176484
Opportunistic salpingectomy in non-gynecological surgeries^37^	Dana Josephy	Interventional	42	Completed	Acceptance of OS among non-gynaecologic surgical patients (aged 45 years or older, or aged 40 years or older with a desire for bilateral salpingectomy) Performance rate of OS among non-gynaecologic surgical patients Duration (in minutes) of salpingectomy Complications up to 30 days after salpingectomy	NCT06032962
Preventing ovarian cancer through opportunistic salpingectomy at the time of colorectal surgery^35^	University of British Columbia	Non-randomized interventional trial	240 of which 120 controls	Recruiting, study (completion estimated in 2026)	The percentage of participants who consented to OS during colorectal surgery The percentage of the participants who accepted OS and who had both fallopian tubes removed The number of added minutes (on average) in the OR to attempt or complete removing both fallopian tubes Incidence of adverse events	NCT05300711

OS, opportunistic salpingectomy; OR, operation room.

### Surgical feasibility

Three studies investigated the surgical feasibility of OS during non-gynaecological surgery^32–34^ and three ongoing clinical trials are registered^35–37^. Two studies reported on bariatric surgery: one conducted sterilization at the time of surgery^32^ and one focused on only the visualization of the fallopian tubes^33^. In the case study of Williams *et al.*^32^ 19 patients underwent sterilization at the time of bariatric surgery (Roux-en-Y gastric bypass (*n* = 11), gastric sleeve (*n* = 8)). No additional incisions or instruments were required. The majority of these patients underwent a bilateral salpingectomy. In one patient, a salpingectomy could not be performed due to pelvic adhesions. The pilot study by Sagmeister *et al.*^33^ found that 25 of 31 patients’ (80.6%) fallopian tubes could be reached and visualized during bariatric surgery. In six patients the visualization was not feasible (19.4%).

In a broader multicentre study, 105 patients aged ≥45 years underwent elective laparoscopic cholecystectomy with OS^34^. The procedure was successfully completed in 98 patients (93.3%). In seven patients, a salpingectomy could not be performed due to the presence of pelvic adhesions (6.7%). A total of 79 OS procedures were performed by a surgeon independently, 19 solely by a gynaecologist and 7 were collaborative efforts between both specialists. No intraoperative or postoperative complications were reported in the above-mentioned studies. On average, surgical procedures were prolonged by 3.5 to 13 min. One patient presented with OC 28 months after cholecystectomy with OS. The initial pathology report indicated normal fallopian tubes. However, upon re-evaluation of the slides using sectioning and extensive examination of the fimbriated end (SEE-FIM), a small focal STIC was identified, which had been previously missed.

The clinical trial (NCT05300711) is investigating the effectiveness of OC prevention through OS at the time of colorectal surgery^35^ and is still recruiting. Trial NCT06312124 is examining the acceptance and uptake of OS among non-gynaecologic surgical patients aged ≥45 years after watching an educational video^36^. Similarly, trial NCT06032962 is assesing acceptance and uptake rates of OS in non-gynaecologic surgical patients aged ≥45 years (or ≥40 years if bilateral salpingectomy is desired), along with salpingectomy duration and complications within 30 days postsurgery^37^. In this trial, OS is performed by a general surgeon.

#### Patients’ perspectives

Three studies reported on patients’ perspectives on OS during non-gynaecological surgery^34,38,39^. Among the counselled patients in a quantitative multicentre study, the majority (62%) accepted OS during elective laparoscopic cholecystectomy^34^. In the preceding study by the same research group^39^, nearly all participants (19 of 20) expressed a willingness to consider OS at the time of cholecystectomy during face-to-face interviews. Seventeen participants favoured the idea, while two patients expressed a need for additional consideration time. Twelve patients were inclined to consent to OS immediately, whereas seven patients were open to an OS but needed more time to make a decision. Most patients desired consultation before surgery to discuss OS with a physician (*n* = 15, 75%); eight of them wanted to discuss OS with their gynaecologist or general practitioner. Of 20 patients, seven expressed a preference to receive information regarding OS at least 2 weeks before their scheduled operation. A significant proportion of the interviewed patients (*n* = 15) indicated that they would find it reassuring to reduce their risk of OC through OS. The majority anticipated that undergoing OS would not adversely affect their sexuality or femininity. Six patients expressed a willingness to incur costs associated with OS.

Cook *et al.*^38^ assessed the willingness to undergo OS during cholecystectomy, hernia repair surgery and bariatric surgery for patients aged 18 to 75 years. An additional handout addressing OC risk and OS was supplied before surgery. Participants were asked to complete a survey by telephone and 37 patients were willing to participate (total of 40; 92.5%). Among the surveyed cohort, 25 patients (67.7%) would desire OS during their scheduled operation, six (16.2%) conveyed a strong interest, while two patients (5.4%) would decline based on a wish to have children. Collectively, all participants expressed the view that both the information on OC risk and the option of OS should be offered to all patients undergoing elective abdominal surgery. The majority of respondents (78.4%, 29 of 37) would not require additional consultation if the procedure was performed by a gynaecologist.

#### Physicians’ knowledge

Physicians’ knowledge on OS was evaluated in two survey studies^40,41^: one among primary care practitioners and surgeons (gynaecological and general)^40^ and one among urologic oncologists^41^. Both studies showed that the vast majority of physicians had no knowledge of the tubal origin of OC and the risk-reducing effect of OS. In the study of Bellamy *et al.*^40^ 12 of 15 surgeons were unaware of the tubal origin. A survey study performed by Sussman *et al.*^41^ revealed that only 14% of the urologic oncologists were aware that salpingectomy reduces the risk of developing OC. Subsequently, Bellamy *et al*. indicated a lack of confidence in discussing OS. Surveyed physicians suggested that written informative resources for physicians and patients, informational lectures and surgical training from a specialist would be beneficial^40^.

#### Cost-effectiveness

The cost-effectiveness of OS during non-gynaecological surgery was evaluated in four studies^42–45^. The studies of Matsuo *et al*. and Hughes *et al*. were both based on a 65% risk reduction following OS. In the cost-effectiveness analysis of Hughes *et al.*^42^ a recursive Markov model was used to determine the effectiveness of OS during non-gynaecological laparoscopic surgery and their effects on the mortality rate of OC. The model used age-adjusted operation rates for appendectomy, cholecystectomy, hernia repair surgery and colon resections. The impact of OS was examined in patients aged >40 years. OS during elective non-gynaecological surgeries could reduce OC deaths by 6.7% in the USA. Taking the additional costs of the procedures into account, the incremental cost-effectiveness ratio (ICER) for OS is around €12 400 (range for the various operations: €6070–€14 200). This is considered cost-effective as it is below the cost-effective threshold of the USA, also referred to as willingness-to-pay^46^. In addition, cost will be saved in the future because fewer patients will be diagnosed with OC. In the utility and cost-effectiveness model of Matsuo *et al.*^43^, the effectiveness of OS during elective laparoscopic cholecystectomy was evaluated. In this model, 5000 patients were allocated to three age cohorts (40, 50 and 60 years of age). OS was associated with a reduction of OC cases (of approximately 39, 36 and 30) and deaths (approximately 12, 14 and 16) in the 40-, 50- and 60-year-old cohorts. A prolonged operation time of 30 min was estimated. The ICER ranged from €10640 to €25 220 in the three age models, therefore, OS at the time of cholecystectomy is considered cost-effective. In the Markov model by Adjei *et al*.^44^, the cost-utility of OS during six common abdominopelvic procedures (hysterectomy, appendectomy, cholecystectomy, gastric bypass, abdominal hernia repair and colectomy) was evaluated in the US female population aged ≥40 years. All six procedures were found to be less costly and more effective with OS than without OS. The adoption of OS was cost-saving (−€1830) and resulted in a gain of +5.01 quality-adjusted life-years (QALYs), with a negative ICER of –€365 per QALY gained, indicating that concurrent OS is cost-saving. Kather *et al.*^45^ evaluated the cost-effectiveness of OS using a state-transition model in prepandemic Germany. When OS was performed during all eligible gynaecological and non-gynaecological abdominal surgeries, the number of eligible patients was 3.5 times higher compared with when it was only performed during hysterectomy or sterilization. Performing OS during all eligible gynaecological and non-gynaecological abdominal surgeries resulted in a 15.3% reduction in OC cases, with an incremental cost-utility ratio (ICUR) of –€6574.60, suggesting cost savings.

## Discussion

This scoping review of the literature regarding OS during non-gynaecological surgery was performed to identify knowledge gaps and to define research priorities needed for the implementation of OS in non-gynaecological surgery. OS is surgically feasible in pilot studies during bariatric surgery and cholecystectomy, resulting in no additional complications, a slight increase of 3.5 to 13 min operation time and a high success rate of salpingectomy during these surgeries. Moreover, this review showed that most patients undergoing non-gynaecological surgery strongly desire information on OS, with the majority of patients expressing an interest to undergo this procedure. Surgeons and urologists often lack sufficient knowledge regarding the tubal origin of OC. Implementation of OS during non-gynaecological surgery would very likely reduce the incidence and thus the mortality rate of OC. Furthermore, this procedure is most likely cost-effective and will prevent healthcare expenditures associated with OC in the future.

This scoping review found that currently OS is rarely offered during non-gynaecological surgery (OS incidence rate of <0.001)^29^. OS was performed during various non-gynaecological procedures, comprising bowel resection or colostomy surgery, breast surgery, cholecystectomy, bariatric surgery and hernia repair surgery. Nonetheless, the degree to which salpingectomy during breast surgery is truly opportunistic is debatable. However, the authors considered OS during breast surgery opportunistic as anaesthesia-related risks had been incurred^29^. One study reported that 56.5% of patients aged 35–75 years undergoing one of three common abdominal surgeries (cholecystectomy, hernia repair surgery, bariatric surgery) would be eligible for OS after reviewing medical charts^28^. However, this is not yet reflected in the numbers of OS conducted during non-gynaecological surgery. Adoption of this primary surgical prevention strategy could be improved by developing and evaluating an implementation strategy tailored to the needs of patients and physicians^47^. Recent research highlighted the importance of a tailored implementation strategy to enhance its impact^48,49^. According to Grol and Wensing’s Change model, a fundamental step for optimal implementation is understanding the factors (facilitators and barriers) that influence OS implementation from the perspectives of patients and physicians^47^, taking social interaction, economic factors and the organizational context into account^49^. Another prominent issue to address is determining which group of patients is suitable for OS (for example surgery type, age, prognoses).

The data on surgical feasibility presented a high success rate between 80.6 and 93.3% of OS during cholecystectomy and bariatric surgery^32,34^. In some cases, OS could not be performed due to pelvic adhesions. The existence of pelvic adhesions is a well known factor negatively influencing the successful completion of OS^50^. Whether OS failure due to adhesions leads to an increase in cancer worry is unknown, representing a knowledge gap. No intra- or postoperative complications were reported in patients who had undergone OS during the previously mentioned surgeries. This aligns with the data obtained among OS during gynaecological surgery^51^. Operating times were slightly longer, ranging from 3.5 to 13 min^32,34^. This can be explained by relocating the operative site from the upper to the lower abdomen, repositioning the patients into Trendelenburg supine position, the repositioning of trocars and even the placement of an additional trocar. Performing an OS more frequently could potentially shorten the operative times, due to the expectance of a steep learning curve. The surgical attainability of OS during colorectal surgery (NCT05300711) is currently under investigation. For other surgery types such as hernia repair surgery and urological surgery, the surgical attainability has not been studied yet.

In the study of Tomasch *et al*., one patient presented with OC 28 months after cholecystectomy with OS. A focal STIC had been previously missed^34^. Whether pathological examination of the fallopian tubes of patients at population risk is needed is still open to debate. Examining the fallopian tubes removed from patients at general risk could enhance the detection of STIC. Nevertheless, the incidence of STIC is anticipated to be extremely low in the general population (<0.01%)^52^. Furthermore, there is no consensus on the optimal method for examining the fallopian tubes in the general population, as pathological review of the fallopian tubes with a SEE-FIM protocol is time-consuming and expensive. However, assessing the fallopian tubes without performing a SEE-FIM protocol could give a false sense of security. Moreover, the histopathological diagnosis of STIC remains challenging, even among specialized pathologists^53,54^. If OS were to be implemented more broadly, the cost-effectiveness and the workload of the pathological examination of the fallopian tubes should be evaluated.

Among patients, 62% consented to OS during elective cholecystectomy^34^, compared with 96% of counselled patients undergoing elective non-obstetric gynaecological surgery^24^. Comparatively, the acceptance rate of OS as a sterilization method was 94% at the time of caesarean section, whereas it was 75% when solely performed as a permanent contraception method^55^. Several factors could explain the lower acceptance rate of OS during non-gynaecological surgery. First, studies of OS in non-gynaecological surgery are limited as this procedure is currently not a de facto standard. Second, counselling for OS in the study of Tomasch *et al.*^34^ was performed by either a surgeon, a gynaecologist or both, while a subset of interviewed patients (8 of 20) expressed a preference for discussing OS with their gynaecologist or general practitioner^39^. Furthermore, no information is available whether acceptance depends upon the type of specialist performing OS. Therefore, the reasons for not accepting OS should be monitored, as this could give tools to improve the quality of counselling.

Physicians’ knowledge was evaluated in two survey studies, both of which showed that the vast majority of physicians had no knowledge of the tubal origin of OC and the risk-reducing effect of OS^40,41^. Subsequently, physicians indicated a lack of confidence in discussing OS and suggested that written informative resources, informational lectures and surgical training from a specialist would be beneficial^40^. Informative resources could be facilitated through the use of a decision aid, which is already available for gynaecological patients^56^. However, it is necessary to assess whether this decision aid can be adapted to meet the needs of non-gynaecological patients. On the other hand, it is necessary to develop strategies to increase knowledge among physicians and ensure proper instruction in surgical techniques^57^.

As increasing evidence supports the advantages of OS, it is important to consider the financial implications. The cost-effectiveness analyses of Hughes *et al.*^42^ and Matsuo *et al.*^43^ suggest that OS during non-gynaecological surgeries as a preventive strategy is most likely cost-effective and will lead to a significant reduction in OC cases in various scenarios; both analyses included patients aged above 40 years. However, an OS could potentially be offered to patients aged above 30 years, therefore, future investigations should include younger patients in their analysis. Furthermore, data on the cost-effectiveness of OS during urological surgeries is missing. As OS has the potential to save a substantial amount of financial costs, research is needed to distinguish which procedures will yield the greatest benefits regarding saving healthcare costs and OC incidence. A limitation of the existing cost-effectiveness analyses is the lack of well defined risk-reducing effects of OS, as there are currently no prospective studies with long-term outcomes; most available evidence comes from retrospective studies. These retrospective studies report varying results regarding the risk-reducing effect of salpingectomy, ranging from no effect—this observation was based on a few incident cases and a relatively short follow-up interval—to a risk reduction of 65–80%^11,15,58,59^. The risk-reducing effect incorporated into both cost-effectiveness analyses can significantly influence the outcomes, highlighting the need for further research in this area. However, as noted in the study by Matsuo *et al.*^43^ in a one-way sensitivity analysis, OS during laparoscopic cholecystectomy remained cost-effective if the associated OC risk reduction was 15% or higher. In addition to healthcare costs, future cost-effectiveness calculations should also consider societal costs, such as absenteeism from work.

A strength of this scoping review lies in providing a complete overview of the currently available literature, attained through a comprehensive and detailed search strategy. Additionally, all studies that reported original data including conference abstracts were included and ongoing clinical trials are mentioned. Corresponding authors of conference abstracts were contacted to enlarge data availability. The search was expanded beyond OS by tubal surgery and sterilization, as OS could be a method of female sterilization. The absence of a quality assessment in this review may be considered a limitation; however, in line with the broad objectives and exploratory nature of this scoping review, conducting such an assessment could detract from the comprehensive mapping of diverse literature types and identification of research gaps. Therefore, transparent reporting and purposeful inclusion of studies with varying quality levels was prioritized to fulfil the objectives. Another limitation of this study lies in its exclusive inclusion of English language literature.

OS during non-gynaecological abdominal surgery could be a powerful prevention strategy that will reduce the incidence and mortality rate of OC, while being cost-effective. However, OS at non-gynaecological surgery has not been widely implemented to date. Therefore, future research should be directed to determine the facilitators and barriers of OS during non-gynaecological abdominal surgery for potential eligible patients and physicians, taking social interaction, economic factors and the organizational context into account.

## Supplementary Material

zrae161_Supplementary_Data

## Data Availability

Data from screening and full-text review are available from the authors upon reasonable request.
