# Supplementary material for: Ovarian cancer risk reduction by salpingectomy during non-gynaecological surgery: scoping review
Source: BJS Open. 2025 Jan 28;9(1):zrae161. doi: 10.1093/bjsopen/zrae161 (PMC11773000; doi:10.1093/bjsopen/zrae161)
Supplement: zrae161_Supplementary_Data [file zrae161_supplementary_data.zip › Supplementary_material.docx]

**Ovarian cancer prevention by salpingectomy during non-gynecological surgery: scoping review**

Charlotte Fisch^1^, Malou Gelderblom^1^, Rosella Hermens^2^, Philip de Reuver^3^, Simon Nienhuijs^4^, Diederik Somford^5^, Joanne de Hullu^1^, Jurgen Piek^2^

1. Department of Obstetrics and Gynecology, Radboud University Medical Centre, Nijmegen, The Netherlands
2. Department of IQ Health, Radboud University Medical Centre, Nijmegen, The Netherlands
3. Department of Surgery, Radboud University Medical Centre, Nijmegen, The Netherlands
4. Department of Surgery, Catharina Hospital, Eindhoven, The Netherlands
5. Department of Urology, Canisius-Wilhelmina Hospital, Nijmegen, The Netherlands
6. Department of Obstetrics and Gynecology, and Catharina Cancer Institute, Catharina Hospital, Eindhoven, The Netherlands

Corresponding author: Charlotte Fisch, Radboud Institute for Health Sciences, Department of Obstetrics and Gynecology, Radboud University Medical Center, PO box 9101, 6500 HB Nijmegen, The Netherlands. Phone number: +31 6 282 424 15 email address: [Charlotte.fisch@radboudumc.nl](mailto:Charlotte.fisch@radboudumc.nl)

<https://orcid.org/0000-0001-6103-3285>

**Supplementary Materials - Index**

| **Supplementary Methods** |  |
| --- | --- |
| Supplementary A: (PRISMA-ScR) Checklist | *pag. 3* |
| Supplementary B: search strategy | *pag. 4* |
| **Supplementary Figures and Tables** |  |
| Supplementary C: Data extraction table | *pag. 8* |
| **References** | *pag. 13* |
|  |  |

**Supplementary Methods**

**Supplementary A: (PRISMA-ScR) Checklist**

Added in a separate file.

**Supplementary B: search strategy**

**PubMed 7 November 2024 (1083 hits)**

(salpingectomy[mesh] OR "Fallopian Tubes/surgery"[MeSH] OR salpingect*[tiab] OR tubectom*[tiab] OR tube excis*[tiab] OR tube remov*[tiab] OR tube resect*[tiab] OR tubal excis*[tiab] OR tubal remov*[tiab] OR tubal resect*[tiab] OR permanent contracept*[tiab] OR definitive contracept*[tiab] OR "Sterilization, Tubal"[Mesh] OR "tubal sterilization"[tiab:~2] OR "tubal sterilisation"[tiab:~2])

AND

("Ovarian Neoplasms/prevention and control"[Mesh] OR "Prophylactic Surgical Procedures"[Mesh] OR "Risk Reduction Behavior"[Mesh] OR nongynecolog*[tiab] OR non-gynecolog*[tiab] OR nongynaecolog*[tiab] OR non-gynaecolog*[tiab] OR opportunisti*[tiab] OR prophylact*[tiab] OR risk reduc*[tiab] OR "cancer prevention"[tiab:~2] OR "cancer preventing"[tiab:~2] OR "Bariatric Surgery"[Mesh] OR "Bariatric Surgery"[tiab:~2] OR "abdominal surgery"[tiab:~2])

**Embase 7 November 2024 (1147 hits)**

(salpingectomy/ OR salpingect*.ti,ab,kf. OR tubectom*.ti,ab,kf. OR tube excis*.ti,ab,kf. OR tube remov*.ti,ab,kf. OR tube resect*.ti,ab,kf. OR tubal excis*.ti,ab,kf. OR tubal remov*.ti,ab,kf. OR tubal resect*.ti,ab,kf. OR uterine tube sterilization/ OR (tubal adj2 (sterilization or sterilization)).ti,ab,kf. OR permanent contracept*.ti,ab,kf. OR definitive contracept*.ti,ab,kf.)

AND

(exp ovary carcinoma/pc OR prophylactic surgical procedure/ OR risk reduction/ OR cancer prevention/ OR nongynecolog*.ti,ab,kf. OR non-gynecolog*.ti,ab,kf. OR nongynaecolog*.ti,ab,kf. OR non-gynaecolog*.ti,ab,kf. OR opportunisti*.ti,ab,kf. OR prophylact*.ti,ab,kf. OR risk reduc*.ti,ab,kf. OR (cancer adj2 prevent*).ti,ab,kf or bariatric surgery/ or ((Bariatric or abdominal) adj2 surgery).ti,ab,kf.)

**Web of Science 7 November 2024 (1257 hits)**

TS=((salpingect* OR tubectom* OR tube NEAR/2 excis* OR tube NEAR/2 remov* OR tube NEAR/2 resect* OR tubal NEAR/2 excis* OR tubal NEAR/2 remov* OR tubal NEAR/2 resect* OR tubal NEAR/2 sterilization OR tubal NEAR/2 sterilisation OR permanent contracept* OR definitive contracept* )

AND

(nongynecolog* OR non-gynecolog* OR nongynaecolog* OR non-gynaecolog* OR opportunisti* OR prophylact* OR risk reduc* OR cancer NEAR/2 prevent* OR Bariatric NEAR/2 surgery OR abdominal NEAR/2 surgery))

**Cochrane library 7 November 2024 (160 hits)**

([mh salpingectomy] OR [mh "Fallopian Tubes - su"] OR salpingect*:ti,ab,kw OR tubectom*:ti,ab,kw OR ("tube" NEAR/2 excis*):ti,ab,kw OR ("tube" NEAR/2 remov*):ti,ab,kw OR ("tube" NEAR/2 resect*):ti,ab,kw OR ("tubal" NEAR/2 excis*):ti,ab,kw OR ("tubal" NEAR/2 remov*):ti,ab,kw OR ("tubal" NEAR/2 resect*):ti,ab,kw OR [mh “Sterilization, Tubal”] OR (tubal NEAR/2 sterilization):ti,ab,kw OR (tubal NEAR/2 sterilisation):ti,ab,kw OR “permanent contracept*”:ti,ab,kw OR “definitive contracept*”:ti,ab,kw)

AND

([mh "Ovarian Neoplasms - pc"] OR [mh "Prophylactic Surgical Procedures"] OR [mh "Risk Reduction Behavior"] OR nongynecolog*:ti,ab,kw OR non-gynecolog*:ti,ab,kw OR nongynaecolog*:ti,ab,kw OR non-gynaecolog*:ti,ab,kw OR opportunisti*:ti,ab,kw OR prophylact*:ti,ab,kw OR ("risk" NEAR/2 reduc*):ti,ab,kw OR (cancer NEAR/2 prevent*):ti,ab,kw OR [mh “bariatric surgery”] OR (Bariatric NEAR/2 surgery):ti,ab,kw OR (abdominal NEAR/2 surgery):ti,ab,kw)

**CINAHL 7 November 2024 (379 hits)**

((MH "salpingectomy") OR (MH "Fallopian Tubes/SU")  OR (TI salpingect* OR AB salpingect*) OR (TI tubectom* OR AB tubectom*) OR (TI "tube excis*" OR AB "tube excis*") OR (TI "tube remov*" OR AB "tube remov*") OR (TI "tube resect*" OR AB "tube resect*") OR (TI "tubal excis*" OR AB "tubal excis*") OR (TI "tubal remov*" OR AB "tubal remov*") OR (TI "tubal resect*" OR AB "tubal resect*") OR (MH "Sterilization, Tubal") OR TI(tubal sterilization or tubal sterilisation) OR AB(tubal sterilization or tuabal sterilisation) OR TI(permanent contracept* OR definitive contracept*) OR AB(permanent contracept* OR definitive contracept*) )
AND

((MH "Carcinoma, Ovarian Epithelial") OR (MH "Ovarian Neoplasms/PC") OR (MH "Surgery, Prophylactic") OR (TI nongynecolog* OR AB nongynecolog*) OR (TI non-gynecolog* OR AB non-gynecolog*) OR (TI nongynaecolog* OR AB nongynaecolog*) OR (TI non-gynaecolog* OR AB non-gynaecolog*) OR (TI opportunisti* OR AB opportunisti*) OR (TI prophylact* OR AB prophylact*) OR (TI "risk reduc*" OR AB "risk reduc*") OR TI "cancer N2 prevent*"  OR  AB "cancer N2 prevent*") OR (MH "Bariatric Surgery") OR TI(Bariatric N2 surgery OR abdominal N2 surgery) OR AB(Bariatric N2 surgery OR abdominal N2 surgery))

**Clinical trials 7 November 2024 (99 hits)**

salpingectomy OR tubectomy

**ICTRP 7 November 2024 (112 hits)**

salpingectomy OR Tubectomy

**Supplementary Figures and Tables**

**Supplementary C: Data extraction table**

| Authors and year | Title | Country of origin | Study design | Study population/ intervention data | Aim | Key outcomes |
| --- | --- | --- | --- | --- | --- | --- |
| N. Adjeia, P. Yehb, A. Haasa, H. Zhaoa, R. Stonec ∙ K.Long Roched, et al.^(1)^  2024 | Opportunistic salpingectomy during gynecologic and non-gynecologic abdominopelvic procedures for ovarian cancer primary prevention: A cost-effectiveness analysis | United States | Conference abstract (Cost-effectiveness analyses) | A markov model to estimate the cost-utility of OS during six common abdominopelvic procedures (hysterectomy, appendectomy, cholecystectomy, gastric bypass, abdominal hernia repair and colectomy) in the female US population aged ≥40 years. | To evaluate the cost-effectiveness and impact of OS during 6 common abdominopelvic procedures on OC costs and overall survival. | - All six procedures were less costly and more effective with OS than   without OS.   - Adoption of OS was cost-saving (−$1,920) and resulted in gained QALYs (+5.01 QALYs), with a negative ICER of $-382.83 per QALY gained. - In all six procedures, OS deemed to be cost saving with a negative ICER. |
| J. Bellamy, A. Page, S. Taylor, M. Tischkowitz, R. Crawford and H. Bolton ^(2)^  2017 | Opportunistic salpingectomy: A survey of knowledge and attitudes in healthcare professionals | United Kingdom | Conference abstract (survey study) | N=36 (21 primary care practitioners and 15 surgeons (gynecologic and general surgeons). | Evaluate knowledge and attitudes of healthcare professionals to OS | - 12 of 15 surgeons were totally unaware of the tubal origin of HGSC. - There was a lack of confidence in discussing or offering OS across all specialties for a variety of reasons. - The majority of professionals commented that written information for healthcare professionals and patients, teaching sessions and/or direct surgical training from a specialist would be helpful. |
| A. M. Cathcart, R. Harrison and J. Luccarelli ^(3)^  2023 | Opportunistic salpingectomy during non-gynaecological surgery in the United States: a population-based retrospective study | United States | Population-based, retrospective, cross-sectional observational study | Discharge data from the National Inpatient Sample (NIS) between 2016 and 2020, n=1,558,020 hospital admissions | To estimate the incidence of  OS during non-gynaecological surgery  at a population level in the USA. | - Between 2016 to 2020, a total of 735 (95% CI 491 to 979) out of 1,558,020 hospital admissions were identified, where bilateral salpingectomy was performed during non-gynaecological surgery. - Hence, 0.05 per cent of all inpatient bilateral salpingectomies were performed during non-gynaecological surgery. - Bilateral salpingectomy was performed simultaneously with bowel resection or ostomy surgery (27.2%, 200/735), breast surgery (8.2%, 60/735), cholecystectomy (7.5%, 55/ 735), bariatric surgery (29.2%, 215/ 735) and hernia repair surgery (19.7%, 145/735). - 85.7 Per cent of bilateral salpingectomies were performed elective during non-gynaecological surgery (630/735). - Procedures were done via laparoscopy (54.4 %, 400/735), robotic-assisted laparoscopy (14.3%, 105/735) or open surgery (31.3%, 230/735). - The median patient age was 37 (interquartile range 34–43) years, the median length of stay was 2 (interquartile range 1–4) days. |
| A. M. Cathcart and R. Harrison ^(4)^  2024 | Opportunistic salpingectomy during non-gynecologic ambulatory surgery in the United States, 2016–2020 | United States | Population-based, retrospective, cross-sectional observational study | All ammbulatory procedures in the National Ambulatory Surgery Sample (NASS) between 1/1/2016 and 12/31/2020 among female patients age 18 and older.  N= 290 389 ambulatory procedures | To estimate the incidence of OS during non-gynecologic ambulatory procedures, to analyze trends in OS incidence over time and assess differences in encounter costs associated with OS. | - The incidence of OS during non-gynecologic surgery was estimated to be between 2,328 and 2,970 cases (95% CI), accounting for 0.9% of all bilateral salpingectomies. - The incidence of OS during non-gynecologic surgery increased over the study period at a quarterly rate of 2.6% (95% CI: 1.1%–4.1%, p < 0.001). - Ambulatory performed procedures were hernia repair (1,885, 71.2 %), cholecystectomy (595, 22.5 %), appendectomy (115, 4.4 %), and other (53, 1.9 %). - Costs for cholecystectomy with OS were $5,067 higher than cholecystectomy without OS (median total encounter charges; $25,940 vs $20,873, p 0.001). |
| I. C. Cook and C. N. Landen^(5)^  2020 | Opportunistic Salpingectomy in Women Undergoing Non-Gynecologic Abdominal Surgery | United States | Conference abstract (institutional review) | 1. Three common non-gynecologic abdominal surgeries (Cholecystectomy, Ventral Hernia Repair and Gastric Weight Loss) charts were examined  - from June 1, 2016 to July 31, 2019 - patients aged 35-75 to evaluate who could benefit from OS.  1. US population data was reviewed for total annual number of each surgery type and reported incidence of ovarian cancer (14,000) including baseline population risk (1.7%). 2. Reduced rate of EOC of 65% after salpingectomy was used. | To estimate the impact that OS  during non-gynecologic procedures would have on the incidence of Epithelial Ovarian cancer (EOC) | - 56.5%, 341 out of 604 cases were eligible for salpingectomy (no history of hysterectomy or tubal sterilisation). - Extrapolating percentage of female patients from the institution undergoing Cholecystectomy (67%), Ventral Hernia Repair (67.5%), and Gastric Weight loss (84%) to the reported total cases per year in the US gave a range for each procedure of: Cholecystectomy (201,000-335,000), Ventral Hernia Repair (236,250-337,500) and Gastric Weight loss (191,520). An estimated 355,255-488,171 patients would be eligible per year. - Assuming 14,000 new EOC cases per year in the US, Baseline population risk of 1.7%, and risk reduction after salpingectomy of 65%, the EOC cases eliminated annually would be between 3,925 and 5,394 cases (28- 38.5% reduced incidence). |
| I. C. Cook, P. T. Hallowell, M. R. Conaway and C. N. Landen ^(6)^  2021 | Patient attitudes toward opportunistic salpingectomy in non-gynecologic surgery | United States | Poster presentation, survey study | N=40 patients aged 18-75 years of a single institution undergoing common non-gynecologic abdominal surgeries (Cholecystectomy, Ventral Hernia Repair and Bariatric Surgery) | To assess the interest of patients undergoing scheduled laparoscopic abdominal surgery for non-gynecologic  indications have regarding OS. | - 40 patients received a one-page handout with information regarding ovarian cancer and OS before surgery. - 37 out of 40 patients were willing to complete a survey (92.5% completion rate). - 100% (37/37) patients felt that the information offered to them regarding ovarian cancer and OS was sufficient. - 100% (37/37) felt it should be offered to all patients undergoing elective abdominal surgery. - 29/37 (78.4%) reported that they would not necessarily need an additional appointment to discuss the procedure if it was to be performed by a gynecologist. - 25/37 (67.6%) patients said they would absolutely want am OS during upcoming g surgery, 6/37 (16.2%) reported strong interest and 2/37 (5.4%) reported they would not be interested because of a child wish. |
| B. N. Hughes, T. J. Herzog, J. Brown, and R. W. Naumann ^(7)^  2022 | Opportunistic Salpingectomy at Time of Nongynecological Elective Procedures Could Reduce Ovarian Cancer-Related Costs and Mortality | Unites States | Cost-effectiveness analyses | A recursive Markov model was developed including age-adjusted rates for appendectomy, cholecystectomy, hernia repair and colon resections. among the effect of OS in patients ≥40 years of age and bilateral salpingo-oophorectomy in patients >50 years of age were analyzed.  The model was used to calculate life expectancy, overall health care costs, and costs of caring for patients with ovarian cancer. | To determine the cost-effectiveness of OS and oophorectomy during non gynecologic laparoscopic procedures and their effects on ovarian-cancer mortality. | - OS during elective non-gynecologic surgeries reduces ovarian cancer deaths by 6.7%. - Bilateral salpingo-oophorectomy in patients older than 50 years of age reduces mortality by 10.0%. - In the US, OS with BSO during cholecystectomy prevents the most deaths at 973, compared to OS alone during colon-cancer surgery, which prevents just 91 deaths per year. Taken the costs of the procedures into account, the incremental cost-effectiveness ratio for OS and BSO is $6,567 (range: $3,874–$9,605). - Considering the cost of caring for patients with OC, OS and BSO produces cost savings. New approved drugs for maintenance therapy could increase the cost savings as high as $296 per capita translating into a health care cost saving of $626 million annually in the US. |
| A. Kather, H. Arefian, M. Hartmann, C. Schneider, I. B. Runnebaum ^(8)^  2024 | Opportunistic salpingectomy for reducing the risk of ovarian cancer: a comprehensive decision-analytic model for clinical and cost-effectiveness in gynecological and general abdominal surgery | Germany | Conference abstract (Cost-effectiveness analyses) | A state-transition model was used to evaluate the cost-effectiveness of four OS strategies in pre-pandemic Germany.  The four strategies were: I) OS during all suitable gynecological and abdominal surgeries, II) OS during all suitable gynecological surgeries, III) OS only during hysterectomy or sterilization, and IV) no OS. | To evaluate te cost-effectiveness of (OS) in Germany. | - The number of eligible patients was 3.5 times higher when OS was performed during all suitable gynecological and abdominal surgeries compared to only during hysterectomy or sterilization. - The reduction in OC cases was 15.3% when OS was performed during all suitable gynecological and abdominal surgeries, with an ICUR of -€6,574.60, indicating potential savings. |
| K. Matsuo, L. Chen, S. Matsuzaki, R. S. Mandelbaum, K. M. Ciesielski, J. P. Silva, et al. ^(9)^  2023 | Opportunistic Salpingectomy at the Time of Laparoscopic Cholecystectomy for Ovarian Cancer Prevention: A Cost-effectiveness Analysis | United States | Cost-effectiveness analyses | A decision analytic model to simulate the sequelae of OS performed at the time of laparoscopic cholecystectomy.  5000 Patients were respectively assigned to 3 age cohorts (40, 50 and 60 years of age). The lifetime risk of ovarian for cancer for these age cohorts was assumed to be 1.17%, 1.09%, and 0.92%. OS was estimated to provide a 65% reduction in the risk of ovarian cancer and to require 30 minutes of additional operative time | To perform a cost-effectiveness analysis to examine the utility  and effectiveness of OS performed at the time of elective laparoscopic cholecystectomy. | - The additional cost of OS at laparoscopic salpingectomy ranged from $1898 to 1978. - In a cohort of 5000 patients allocated to 3 age cohorts (40, 50 and 60 years of age). OS was associated with a reduction of OC cases (39, 36, and 30) and deaths (12,14 and 16) in the age 40–, 50–, and 60-year-old cohorts, respectively. - OS was cost-effective, with incremental cost-effectiveness ratio of $11,162 to 26,463 in the 3 age models. - In a probabilistic sensitivity analysis, incremental cost-effectiveness ratio for OS were less than $100,000 per quality-adjusted life-years in 90.5% or more of 1000 simulations. |
| H. Sagmeister, D. Pucher, S. Oswald, F. Tadler, J. Strutzmann and K. Tamussino ^(10)^  2023 | Might prophylactic salpingectomy be possible during bariatric surgery? (Can we see the tubes?) | Austria | Conference abstract (pilot study) | N=31 bariatric surgery patients | To evaluate whether the tubes could be visualized during bariatric surgery. | - In 25 out of 31 patients (81%) the adnexa could be visualized and reached - In one patient only one side of the adnexa was visible - It took on average 3.5 minutes additional (range 1–8 min). |
| R. D. Sussman, C. J. Han, D. Marchalik, F. L. Carvahlo, M. F. Davis, L. A. Richter, et al. ^(11)^  2018 | To oophorectomy or not to oophorectomy: Practice patterns among urologists treating bladder cancer | United States | Survey study | N= 159 urologic oncologists | To assess the knowledge base and current  practice patterns of urologic oncologists regarding  management of the gynecological organs at the time of radical cystectomy. | - 14% of the urologist were aware that salpingectomy alone reduces the risk of ovarian cancer, 83.9% of the urologist responded that it had no effect on the risk of ovarian cancer and 1.9% responded that it increased the risk of ovarian cancer. - Respectively 95%, 66%,26% and 39.7% of the urologist were aware that bilateral salpingo-oophorectomy increases the risk of osteoporosis, cardiovascular disease, all-cause mortality and of the decline in cognitive function. |
| G. Tomasch, B. Bliem, M. Lemmerer, S. Oswald, S. Uranitsch, E. R. Greimel, et al.^(12)^  2018 | Would women accept opportunistic (prophylactic) salpingectomy at the time of non gynecologic surgery to prevent development of ovarian cancer? | Austria | Qualitative interview study | N=20  Patients ≥45 years scheduled for elective laparoscopic cholecystectomy for a  benign indication | To assess whether it would be worthwhile to plan a trial actually offering salpingectomy at the time of laparoscopic cholecystectomy. | - Patients were interview by a clinical psychologist who was not a member of the treatment team. - 19 out of the 20 patients indicated they were open to the idea of an OS at the time of cholecystectomy; 17 patients thought it is a good idea, 2 patients would want to consider it further. - 12 Patients would agree to the OS straightaway, 7 would need more time for making a decision but would not be averse. - One patient would decline OS. - Nine patients had not thought about ovarian cancer and did not consider themselves informed about the disease. Seven patients had concerns about ovarian and other cancers. 15 patients would find it reassuring to reduce ovarian cancer risk. - 17 and 16 patients, respectively thought salpingectomy would not have an impact on femininity or sexuality. - 15 patients would like to discuss OS with a physician before the procedure, 8 patients would like to talk to their family doctor or gynecologist. - 7 patients would like the required information 2 weeks or more before the surgery to make a decision. - 6 patients might be willing to pay extra for the additional procedure |
| G. Tomasch, M. Lemmerer, S. Oswald, S. Uranitsch, C. Schauer, A. M. Schütz, et al. ^(13)^  2020 | Prophylactic salpingectomy for prevention of ovarian cancer at the time of elective laparoscopic cholecystectomy | Austria | Pilot study | Six centers participated  N=105 patients  Inclusion criteria:   - ≥45 years - Elective procedure - Childbearing completed - knowledge of German sufficient to complete questionnaires | To evaluate the feasibility and short-term complications of  prophylactic salpingectomy in patients ≥ 45 years undergoing non-emergency laparoscopic cholecystectomy for benign indications. | - 105 patients consented to concomitant prophylactic salpingectomy. Mean age 55 (range 42–79) years, Median parity 2 (range 0–4). - The rate of acceptance of attempted salpingectomy was 62 % - Salpingectomy was completed in 98 out of 105 patients (93.3%) - In 7 out of 105 patients salpingectomy was not possible as adhesions precluded easy access to, or visualization of, the tubes. Age and BMI did not differ between patients who did and those who did not undergo prophylactic salpingectomy. - The median additional time required for salpingectomy was 13 min. - In 32 out of 105 patients a new device was used for salpingectomy - In the majority, 89 out of 105 patients, the trocars were not repositioned. - In 79 cases the surgeon performed salpingectomy, in 19 cases the gynecologist and in 7 cases both. - No intraoperative or postoperative complications were reported attributable to salpingectomy. - One patient presented 28 months after surgery with ascites and peritoneal carcinomatosis owing to high-grade serous carcinoma. The initial pathology report had described normal tubes; re-evaluation of the slides showed a small, focal STIC. |
| B. Williams ^(14)^  2018 | Concurrent Bilateral Salpingectomy for Sterilisation at the Time of Bariatric Surgery | United States | Conference abstract | N=19 cases of planned bariatric and sterilisation procedures between September 2011 and April 2018 | To demonstrate the safety and feasibility of performing bilateral salpingectomy for sterilisation at the time of  a bariatric operation. | - 19 patients underwent sterilisation procedures at the time of minimally invasive bariatric surgery (11 Roux- en-Y gastric bypass and 8 sleeve gastrectomy), with the vast majority receiving bilateral salpingectomy. - No additional incisions or instruments were required. - Average age body mass index was 46.3 kg/m2. - Average age 39.4 years. - >50% (10/19) of the patients had a history of prior abdominal gynecologic surgery, predominately C-section. - The average additional time required for bilateral salpingectomy was 8.1 minutes. - 1 sterilisation procedure could not be performed, due to dense pelvic adhesions. - No perioperative complications were reported. |

**References**

1. Adjei N, Yeh P, Haas A, Zhao H, Stone R, Roche KL, et al. Opportunistic salpingectomy during gynecologic and non-gynecologic abdominopelvic procedures for ovarian cancer primary prevention: A cost-effectiveness analysis. Gynecologic Oncology. 2024;190(Supplement 1):S26-S7.

2. Bellamy J, Page A, Taylor S, Tischkowitz M, Crawford R, Bolton H. Opportunistic salpingectomy: A survey of knowledge and attitudes in healthcare professionals. International Journal of Gynecological Cancer. 2017;27(Supplement 4):326.

3. Cathcart AM, Harrison R, Luccarelli J. Opportunistic salpingectomy during non-gynaecological surgery in the United States: a population-based retrospective study. Br J Surg. 2023.

4. Cathcart A, Harrison R. Opportunistic salpingectomy during non-gynecologic ambulatory surgery in the United States, 2016-2020 12. Gynecologic Oncology. 2024;190(Supplement 1):S181-S2.

5. Cook IC, Landen CN. Opportunistic Salpingectomy in Women Undergoing Non-gynecologic Abdominal Surgery. Gynecologic Oncology. 2020;158(1):e2.

6. Cook IC, Hallowell PT, Conaway MR, Landen CN. Patient attitudes toward opportunistic salpingectomy in non-gynecologic surgery. European Journal of Gynaecological Oncology. 2021;42(2):391-2.

7. Hughes BN, Herzog TJ, Brown J, Naumann RW. Opportunistic Salpingectomy at Time of Nongynecologic Elective Procedures Could Reduce Ovarian Cancer–Related Costs and Mortality. Journal of Gynecologic Surgery. 2022;38(1):43-8.

8. Kather A, Arefian H, Hartmann M, Schneider C, Runnebaum IB. Opportunistic salpingectomy for reducing the risk of ovarian cancer: a comprehensive decisionanalytic model for clinical and cost-effectiveness in gynecological and general abdominal surgery. Geburtshilfe und Frauenheilkunde. 2024;84(10):e156-e7.

9. Matsuo K, Chen L, Matsuzaki S, Mandelbaum RS, Ciesielski KM, Silva JP, et al. Opportunistic Salpingectomy at the Time of Laparoscopic Cholecystectomy for Ovarian Cancer Prevention: A Cost-effectiveness Analysis. Annals of Surgery. 2023;277(5):E1116-E23.

10. Sagmeister H, Pucher D, Oswald S, Tadler F, Strutzmann J, Tamussino K. Might prophylactic salpingectomy be possible during bariatric surgery? (Can we see thetubes?). Geburtshilfe und Frauenheilkunde. 2023;83(4):e8-e9.

11. Sussman RD, Han CJ, Marchalik D, Carvahlo FL, Davis MF, Richter LA, et al. To oophorectomy or not to oophorectomy: Practice patterns among urologists treating bladder cancer. Urol Oncol. 2018;36(3):90.e1-.e7.

12. Tomasch G, Bliem B, Lemmerer M, Oswald S, Uranitsch S, Greimel ER, et al. Would women accept opportunistic (prophylactic) salpingectomy at the time of nongynecologic surgery to prevent development of ovarian cancer? Surgery. 2018;164(5):931-4.

13. Tomasch G, Lemmerer M, Oswald S, Uranitsch S, Schauer C, Schütz AM, et al. Prophylactic salpingectomy for prevention of ovarian cancer at the time of elective laparoscopic cholecystectomy. Br J Surg. 2020;107(5):519-24.

14. Williams B. Concurrent Bilateral Salpingectomy for Sterilization at the Time of Bariatric Surgery. Surgery for Obesity and Related Diseases. 2018;14(11 Supplement):S150.
